# Flow‐Active Liquid Marbles as Microreactors for Photocatalytic Micromotors

**DOI:** 10.1002/smll.202505439

**Published:** 2025-09-12

**Authors:** Anthony Jesús Martínez, Majid Basharat, Shuqin Chen, Samuel Sánchez, Katherine Villa

**Affiliations:** ^1^ Institute of Chemical Research of Catalonia (ICIQ‐CERCA) The Barcelona Institute of Science and Technology (BIST) Av. Països Catalans, 16 Tarragona E‐43007 Spain; ^2^ Departament de Química Física i Inorgànica Universitat Rovira i Virgili Marcel.lí Domingo 1 Tarragona 43007 Spain; ^3^ Institute for Bioengineering of Catalonia (IBEC) The Barcelona Institute for Science and Technology (BIST) Baldiri i Reixac 10–12 Barcelona 08028 Spain; ^4^ Catalan Institute for Research and Advanced Studies (ICREA) Psg. Lluis Companys 23 Barcelona 08010 Spain

**Keywords:** active matter, liquid marbles, marangoni flow, photocatalysis, self‐propelled micromotors

## Abstract

Self‐propelled micromotors have shown promise for applications in environmental remediation, sensing, and biomedicine. However, assessing their performance in realistic, 3D microenvironments with dynamic boundaries and complex topography remains a key challenge. Achieving controlled motion and enhanced reactivity under such confinement is critical for both technological applications and fundamental studies on active matter. Here, the integration of light‐driven micromotors with liquid marbles is presented, which are gas‐permeable droplets encased by hydrophobic particles that act as dynamic, flow‐active microreactors. By tuning the coverage of the particulate shell, partially covered liquid marbles are developed that exhibit robust evaporation‐induced flows, increasing the average micromotor velocity by approximately threefold compared to sessile droplets. Under illumination, photocatalytic self‐propulsion provides an additional velocity component and promotes micromotor dispersion. The combined circulation enhances mass transfer, guiding micromotor accumulation and transport while providing an optical transparent, soft‐confinement platform for studying active particles and confined catalytic reactions.

## Graphical abstract

Light‐powered micromotors inside partially covered liquid marbles couple photocatalysis with flow‐driven transport, achieving higher mobility and enhanced mass transfer. This soft, transparent confinement platform offers a new route to study active matter and catalytic processes at curved liquid–air interfaces.

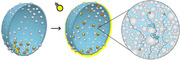

## 1 Introduction

Micro/nanomotors (MNMs) are micro/nanoscale devices capable of converting external energy into autonomous motion, offering new paradigms in targeted transport,^[^ ^1^, ^2^, ^3^ ^]^ cargo delivery,^[^ ^4^, ^5^ ^]^ environmental remediation,^[^ ^6^ ^]^ and microscale catalysis.^[^ ^7^, ^8^ ^]^ As the field evolves,^[^ ^9^ ^]^ the interplay between propulsion and microenvironment becomes increasingly important, not only for real‐world operation but also for fundamental understanding of active matter.^[^ ^10^ ^]^ One of the critical challenges lies in achieving effective motor navigation and chemical reactivity within confined, 3D spaces that better mimic complex natural environments.

Confinement influences the behavior of the MNMs by altering hydrodynamic interactions, electrostatic forces, diffusiophoretic gradients, and particle–boundary interactions.^[^ ^11^ ^]^ These effects become especially prominent near structural features such as grooves, microchannels, steps, and diverse topographic structures,^[^ ^12^ ^]^ which can induce clustering,^[^ ^13^ ^]^ directional alignment,^[^ ^14^ ^]^ trajectory rectification, or speed modulation.^[^ ^15^ ^]^ External fields, *e.g*., electromagnetic, acoustic, or chemical gradients,^[^ ^16^, ^17^, ^18^ ^]^ have also been employed to impose confinement and guide motion behavior. However, most studies have focused on rigid geometries or specialized materials, limiting their adaptability to soft or reconfigurable systems.

A wide variety of soft confinements, such as vesicles,^[^ ^19^, ^20^, ^21^ ^]^ microdroplets,^[^ ^22^ ^]^ emulsions^[^ ^23^ ^]^ and microfluidics compartments, have been explored for studying active matter. In contrast, liquid marbles (LMs) consist of liquid droplets encapsulated by hydrophobic particles, forming a porous, gas‐permeable, and mechanically stable shell that defines a freestanding, deformable 3D domain without requiring a substrate.^[^ ^24^ ^]^ This architecture differs fundamentally from fully enclosed systems such as vesicles or isotropic droplets: the partial shell coverage and curved liquid–air interface introduce a range of interfacial phenomena, including gas exchange, localized evaporation, and porous liquid–air and liquid–solid boundaries. Capillary effects,^[^ ^25^ ^]^ arising from liquid–liquid and liquid–particle attractive forces, govern LM stability by maintaining the spherical shape, promoting interfacial jamming of the particle shell to preserve integrity, and minimizing substrate contact while retaining non‐wetting properties.^[^ ^26^ ^]^ Together with surface‐tension–driven flows and curvature‐enhanced chemical gradients, these effects can sustain long‐lived Marangoni circulation and strongly influence particle dynamics within the marble. Previously, LMs have been used in applications ranging from sensing and cell culture to compartmentalized reactions.^[^ ^27^, ^28^, ^29^ ^]^ However, their potential to host and influence micromotor behavior remains underexplored.

In this work, we investigate LMs as adaptive, soft confinement systems for hosting light‐driven micromotors. We examine how variations in shell coverage influence internal flow generation and micromotor behavior within LMs. By characterizing two distinct marble architectures, including completely covered (CCLMs) and partially covered LMs (PCLMs), we reveal how internal topology, fluid dynamics, and photocatalytic micromotors interact under semi‐enclosed conditions. Notably, micromotors preferentially accumulate near the internal liquid–air interface, where their trajectories are influenced by surface topography and internal flow fields. This platform enables the study of how dynamic confinement shapes micromotor transport and photocatalytic performance, establishing LMs as promising systems for studying active matter and confined photocatalysis in deformable microenvironments.

## 2 Results and Discussion

### 2.1 Liquid Marble Confinement and Structural Characterization

**Scheme 1:**
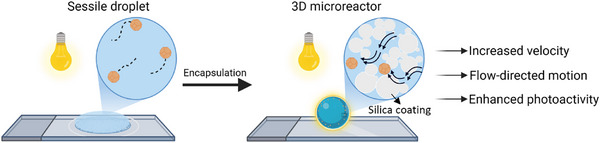
Schematic representation of Cu_2_O micromotors in two different environments: a sessile droplet (left) and a liquid marble (right).

The motion behavior of self‐propelled MNMs is typically studied in sessile droplets on glass slides, where confinement arises from substrate interactions and evaporation‐driven flows. In contrast, LMs provide a gas‐permeable confinement environment with tunable interfacial properties and hydrophobicity.^[^ ^21^ ^]^ Their unique architecture enables the study of micromotor dynamics under fully 3D, non‐wetting, and structurally heterogeneous confinement. As shown in **Scheme** **1**, LMs are defined by a porous particulate shell that creates a semi‐enclosed domain with transport properties distinct from those of sessile droplets. This unique confinement promotes flow‐influenced micromotor motion at higher velocities than typical substrates, and improved mass‐transfer processes.

**Figure 1:**
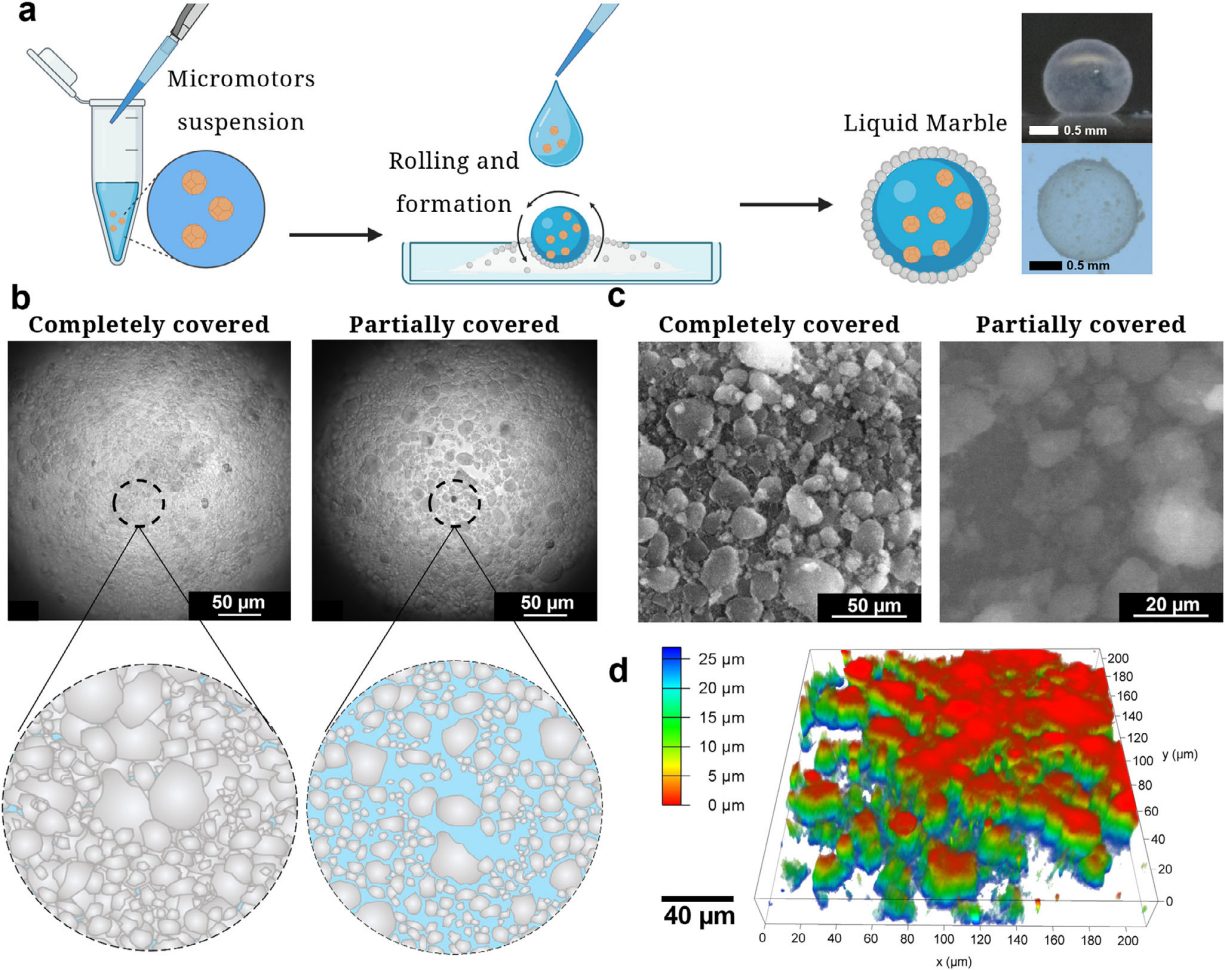
Fabrication and structural characterization of CCLMs and PCLMs. a) Schematic illustration of the LMs formation by rolling aqueous Cu_2_O micromotor suspensions over a bed of hydrophobic fumed silica nanoparticles. b) Inverse bright‐field microscopy images showing the internal surface of a CCLM (left) and a PCLM (right). Insets display schematic representation of both marble types with the water‐air interfaces colored in blue. c) ESEM images of shell morphology for CCLMs (left) and PCLMs (right). d) 3D fluorescence Z‐scan reconstruction of a FITC‐labeled PCLM shell, showing structural heterogeneity including pores and discontinuities.

As illustrated in **Figure** **1**a, LMs were obtained by rolling an aqueous suspension of MNMs over a hydrophobic fumed silica bed, generating a porous shell by adjusting the rolling conditions. Characterization of fumed silica particles and Cu_2_O micromotors used in this work is presented in Figures S1 and S2 (Supporting Information). To characterize the internal architecture of LMs, we performed optical and microscopic analyses focused on their interfacial topology. Inverse bright‐field microscopy enabled direct visualization of the internal liquid–solid interface, made possible by the optical transparency of the hydrophobic silica shell (Figure 1b). By tuning the extent of silica coverage during marble formation, we reproducibly obtained two distinct LMs, including CCLMs and PCLMs. As previously reported, the degree of surface coverage has a direct influence on LMs properties, including permeability, mechanical integrity, and interfacial behavior.^[^ ^30^, ^31^ ^]^ In both cases, the formation of metastable wetting states is governed by surface tension, consistent with the Cassie–Baxter model that describes heterogeneous wetting on composite interfaces.^[^ ^32^ ^]^

Environmental scanning electron microscopy (ESEM) revealed pronounced structural differences between CCLMs and PCLMs. CCLMs displayed densely packed, multilayered silica shells with limited porosity, while PCLMs featured micrometer‐scale pores and scattered silica nanoparticles at the liquid–air interface (Figure 1c). The reduced resolution in PCLMs is caused by pressure fluctuations from water evaporation in the vacuum chamber during ESEM imaging. To further visualize the internal topology, we performed 3D fluorescence microscopy using FITC‐labeled silica particles. The reconstructed volume (200 × 200 × 25 µm^3^) revealed a heterogeneous landscape composed of gaps, grooves, and step‐like features ranging from 10 to 50 µm (Figure 1d). These topographical features were distributed throughout the marble interior and often included large discontinuities containing embedded silica aggregates. This pronounced topographical heterogeneity is expected to influence local flow fields and establish preferential pathways for micromotor motion along the internal interface.

### 2.2 Evaporation‐Induced Microflows in LMs

To probe the presence of internal fluid motion, we introduced 1.5 µm polystyrene (PS) particles as passive tracers into both CCLMs and PCLMs. In CCLMs, the tracers exhibited only Brownian motion, indicating the absence of organized flow near the internal surface. In contrast, tracers in PCLMs followed long, coherent trajectories along the internal interface, often aligned with the spatial distribution of silica aggregates (Video S1, Supporting Information). These observations suggest that the exposed regions of the air–liquid interface in PCLMs support internal hydrodynamic flows, potentially driven by evaporation. To confirm this, we enclosed PCLMs in a sealed chamber to avoid evaporation and found that tracer motion was completely suppressed (Video S2, Supporting Information), confirming the evaporation‐dependent nature of the flows. Importantly, the tracer particles remained confined to a narrow region adjacent to the interface, with no motion detected in the bulk liquid. This localization was further confirmed using fluorescently labeled tracers, which revealed that active transport occurred exclusively along the inner surface of the marble (Video S3, Supporting Information).

While both CCLMs and PCLMs undergo evaporation, internal flows were only observed in PCLMs, indicating that exposure of the liquid–air interface is a key requirement for flow generation. Based on our observations and prior reports on sessile droplets,^[^ ^33^ ^]^ we attribute these flows to evaporation‐induced Marangoni effects at the liquid–air interface. Other contributions, such as buoyancy‐driven^[^ ^34^ ^]^ or thermally induced convection,^[^ ^35^ ^]^ cannot be fully excluded, though they are more likely to influence the bulk phase and typically exhibit uniform, unidirectional flow patterns.^[^ ^35^, ^36^ ^]^ In contrast, the flows observed in PCLMs were dynamic and often vortical, a characteristic feature of surface‐tension‐driven Marangoni circulation.^[^ ^37^, ^38^ ^]^

To elucidate the physical origin of the internal flows observed in PCLMs, we quantified the relative contributions of surface tension and buoyancy‐driven effects using the dimensionless Marangoni (*Ma*) and Rayleigh (*Ra*) numbers. The Marangoni number, which captures flow generation due to surface tension gradients, was calculated using Equation (1), where *|dγ/dT|* represents the variation of surface tension with temperature, *ΔT* is the interfacial temperature gradient, *µ* is water viscosity, *α* is water thermal diffusivity, and *L* is PCLM diameter. *ΔT* was measured through thermographic analysis (Figure S3, Supporting Information), which exhibited temperature changes in the same range along the whole interface. Except for *ΔT* and *L* (Figure S4, Supporting Information), which were measured experimentally, all parameters correspond to known values for water.^[^ ^39^ ^]^ Similarly, the Rayleigh number (Equation 2) was also estimated to characterize buoyancy‐driven convection, with *β* as the thermal expansion coefficient, *ρ* is the density of water, and *g* is gravitational acceleration. The ratio *Ma*/*Ra* (Equation 3) provides a metric for comparing the strength of surface‐tension‐driven flows to those governed by natural convection.^[^ ^33^, ^39^ ^]^ For the PCLMs studied, the calculated values were *Ma* = 710.17 and *Ra* = 35.21, resulting in a *Ma/Ra* ratio of 20.17. This indicates that surface‐tension‐driven Marangoni flows are over 20 times stronger than buoyancy‐driven convection in these conditions, and thus represent the flow dominant mechanism.

(1) $$\mathrm{Ma}=\left|d\gamma /\mathrm{dT}\right|\Delta TL/\mu \alpha$$

(2) $$Ra=\beta \rho g\Delta TL^{3}/\mu \alpha$$

(3) $$\mathrm{Ma}/\mathrm{Ra}=\left|d\gamma /\mathrm{dT}\right|/\beta \rho g\Delta TL^{2}$$

**Figure 2:**
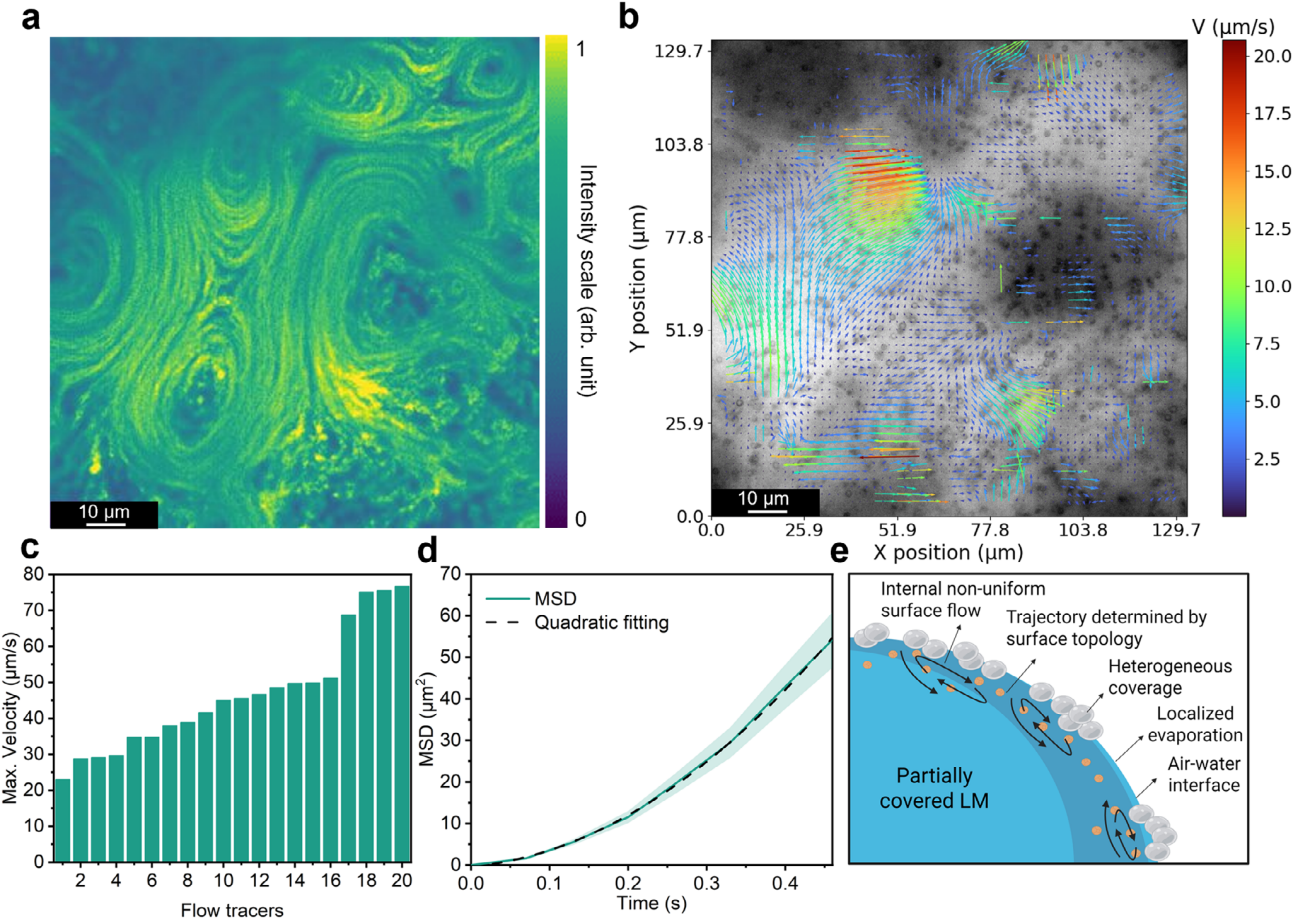
Characterization of interfacial flow in PCLMs with PS tracer particles. Density maps (a) and PIV analysis (b) of PS tracers (0.28% w/v in water). The average pixel intensity was calculated over a 10 s video and visualized using the viridis colormap. PIV analysis indicates the flow field distribution. c) Maximum instant velocities calculated from the tracking of individual PS particles in PCLM (0.028% w/v in water). d) MSD and fitting plot PS particles (*n* = 20). The shaded area represents the measurement error, while the fitting parameters and associated errors are provided in Table S1 (Supporting Information). e) Schematic representation of PCLMs loaded with a suspension of 1.5 µm PS beads, illustrating flow confinement near the internal liquid–air interface and tracer alignment along surface‐guided trajectories.

Interfacial flows patterns were further analyzed using PS tracer particles. Density maps (**Figure** **2**a) revealed Marangoni flows, with high‐intensity regions aligning with the marble gaps. Particle velocimetry analysis (PIV) (Figure 2b) showed a heterogeneous flow field, reaching velocities up to 20 µm s^−1^ in uncovered regions and decreasing near silica‐covered areas of the PCLM.

Additionally, a less concentrated sample was used for single tracer particle tracking. The average tracer velocity in PCLMs was 15.09 µm s^−1^, with some PS tracers reaching up to 80 µm s^−1^ instant velocity along preferred pathways (Figure 2c). As shown in Figure 2d, particle trajectories were analyzed by calculating the mean squared displacement (MSD) over time, as defined in Equation (4), where *Δt* is the time interval and *x_i_* represents the position of the particle at initial time Δt, *i* and *t* represent the dimensional index and time, respectively.^[^ ^40^ ^]^ After plotting the MSD values obtained for these tracers, their behavior is fitted according to Equation (5), where *v* is mean velocity and *D_t_* is the diffusion coefficient.^[^ ^41^ ^]^

(4) $$\begin{array}{ccc} MSD\left(\Delta t\right) & = & \langle {\left(x_{i}\left(t+\Delta t\right)-x_{i}\left(t\right)\right)}^{2}\rangle \\ & & \;\left(i=2\;\mathrm{for}\;\mathrm{two}-\mathrm{dimensional}\;\mathrm{analysis}\right) \end{array}$$

(5) $$\begin{array}{c} MSD=4D_{t}t+\nu^{2}t^{2} \end{array}$$

These velocities are significantly higher than those observed in sessile droplets, where Marangoni flows are typically limited to ∼1 µm s^−1^.^[^ ^42^ ^]^ According to Diddens et al.,^[^ ^43^ ^]^ Marangoni flows in sessile droplets are commonly suppressed by trace amounts of surface‐active contaminants, which act as surfactants and reduce interfacial tension gradients. Remarkably, concentrations as low as 300 molecules µm^−^^2^ can decrease flow speeds by two orders of magnitude.^[^ ^44^ ^]^ In PCLMs, however, this suppression appears to be mitigated. Unlike sessile droplets, PCLMs are encased in a porous, superhydrophobic silica shell rather than resting on solid substrate, greatly reducing contact with external surfaces where contaminants typically accumulate. These findings suggest that the unique interfacial environment in PCLMs may overcome a key limitation of sessile droplets, enabling sustained, robust flow generation for enhanced transport.

In addition to the enhanced velocities and flow patterns, we consistently observed that tracer particles remained confined near the internal liquid–air interface. Figure 2e illustrates this characteristic near‐surface motion. Such localization can be rationalized using the classical Derjaguin–Landau–Verwey–Overbeek (DLVO) theory, which describes the balance of forces in passive colloidal suspensions such as PS beads. Although both the tracers (zeta potential of −21.9 mV) and the silica shell carry net negative surface charges, long‐range electrostatic repulsion is offset by short‐range van der Waals attraction, resulting in a secondary energy minimum that loosely anchors the particles near the interface.^[^ ^45^ ^]^

In complex environments such as PCLMs, however, this behavior may be further modulated by nanoscale surface roughness, heterogeneous charge distribution, and local hydrodynamic forces.^[^ ^46^, ^47^ ^]^ For colloids in flows, stability depends on the type of flow, which can vary from simple shear to vortical fields.^[^ ^48^ ^]^ According to Varma et al.^[^ ^49^ ^]^ such combined effects can stabilize tracer positioning along specific regions of the interface, creating preferential pathways for directed motion. In micromotors, interparticle interactions and DLVO forces are further coupled with self‐generated chemical gradients and collective effects, making them key contributors to aggregation, confinement within flowing regions, and localized catalytic activity.

### 2.3 Micromotor Motion Dynamics Within LMs

**Figure 3:**
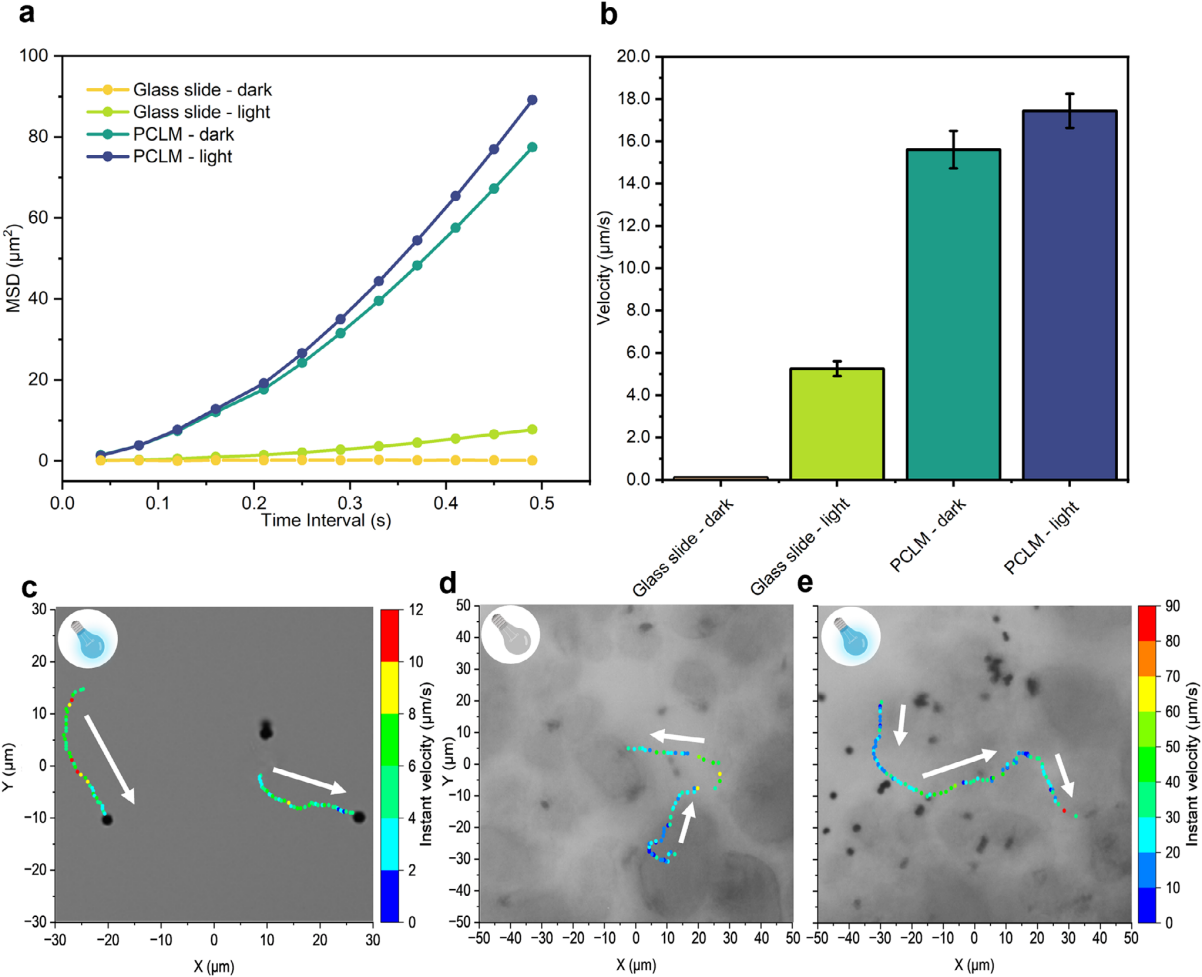
Cu_2_O micromotors behavior in sessile droplets (glass substrate) and PCLMs under dark and light conditions (440 nm). a) MSD plots of micromotors (*n* = 20) under four conditions; glass‐dark, glass‐light, PCLM‐dark, and PCLM‐light. b) Average velocities for each condition, showing significant speed enhancement in PCLMs under illumination. Error bars indicate standard deviation of the mean (*n* = 3). c) Velocity‐colored trajectories of two micromotors recorded on a glass slide under visible light (arrows indicate direction of the motion). d) Velocity‐colored trajectories of micromotors inside PCLMs under dark and under visible light (e), superimposed on bright‐field images, and alignment with flow‐guided paths at the liquid–air interface.

Having confirmed robust interfacial flows in PCLMs, we next investigated their effect on the motion of photoactive Cu_2_O micromotors, which propel in water by light‐driven photocatalytic mechanisms.^[^ ^50^ ^]^ To systematically compare micromotor motion dynamics under different conditions, we quantified their MSD, average velocity, and trajectory behavior in four environments: a glass substrate (sessile droplet) and PCLMs, each tested under light and dark conditions. As shown in **Figure** **3**a, the movement of Cu_2_O micromotors on a glass slide displayed a clear transition in MSD from linear to quadratic fitting upon light activation (Video S4, Supporting Information), consistent with photocatalytic propulsion. In contrast, when the Cu_2_O micromotors were inside PCLMs, they exhibited persistent motion under both dark and light irradiation conditions, indicating that confinement‐enhanced flow supports sustained displacement even without illumination.

Velocity measurements (Figure 3b) revealed that Cu_2_O micromotors in PCLMs reached average speeds above 15 µm s^−1^, approximately threefold higher than those on a glass slide (5.25 µm s^−1^). Instant velocity analysis and representative 2D trajectories (Figure 3c–e) further confirmed the strong effect of confinement and flow on micromotor displacement. Even in the absence of light, Cu_2_O micromotors confined within PCLMs followed long trajectories (Video S5, Supporting Information), driven by the internal Marangoni flows described earlier. In these regions, instantaneous velocities reached up to 90 µm s^−1^, while the average velocity across 20 tracked micromotors was 15.61 µm s^−1^ in dark conditions and further enhanced under illumination, with average velocities increasing up to 17.43 µm s^−1^. In many cases, Cu_2_O micromotors repeatedly followed similar trajectories, dictated by flow patterns shaped by the topography of the silica shell, as described earlier. Additionally, single‐particle tracking was used to quantify the number of micromotors following repeated pathways and their average path length over a 10 s video (Figure S5, Supporting Information). These results suggest a dual‐mode propulsion mechanism: photocatalytic activity provides autonomous motion, while interfacial Marangoni flows assist in propelling Cu_2_O micromotors along a non‐uniform flow field. The curved geometry and soft confinement of the PCLM maintain both flow and mobility over time, enabling coordinated micromotor trajectories rarely observed in open or rigid systems.

Rather than dispersing randomly, Cu_2_O micromotors remain aligned with the internal interface, with some of them navigating through topographically defined pathways. This behavior reflects a soft reversible form of confinement, where flow gradients and local structural features cooperatively bias particle motion. In addition, photocatalytic micromotors generate solute gradients that could be influenced by proximity to curved interfaces, as suggested in prior studies on phoretic systems.^[^ ^13^, ^14^ ^]^ Such curvature‐induced modulation might reinforce propulsion asymmetry, although this effect has not been directly measured here and remains a hypothesis for future investigation. Finally, the absence of rigid boundaries allows for uninterrupted circulation, enhancing both displacement and residence time near reactive interfaces.^[^ ^51^, ^52^ ^]^ This continuous, surface‐guided transport is therefore expected to contribute to the improved distribution of active particles and overall photocatalytic performance within the PCLM microreactor.

**Figure 4:**
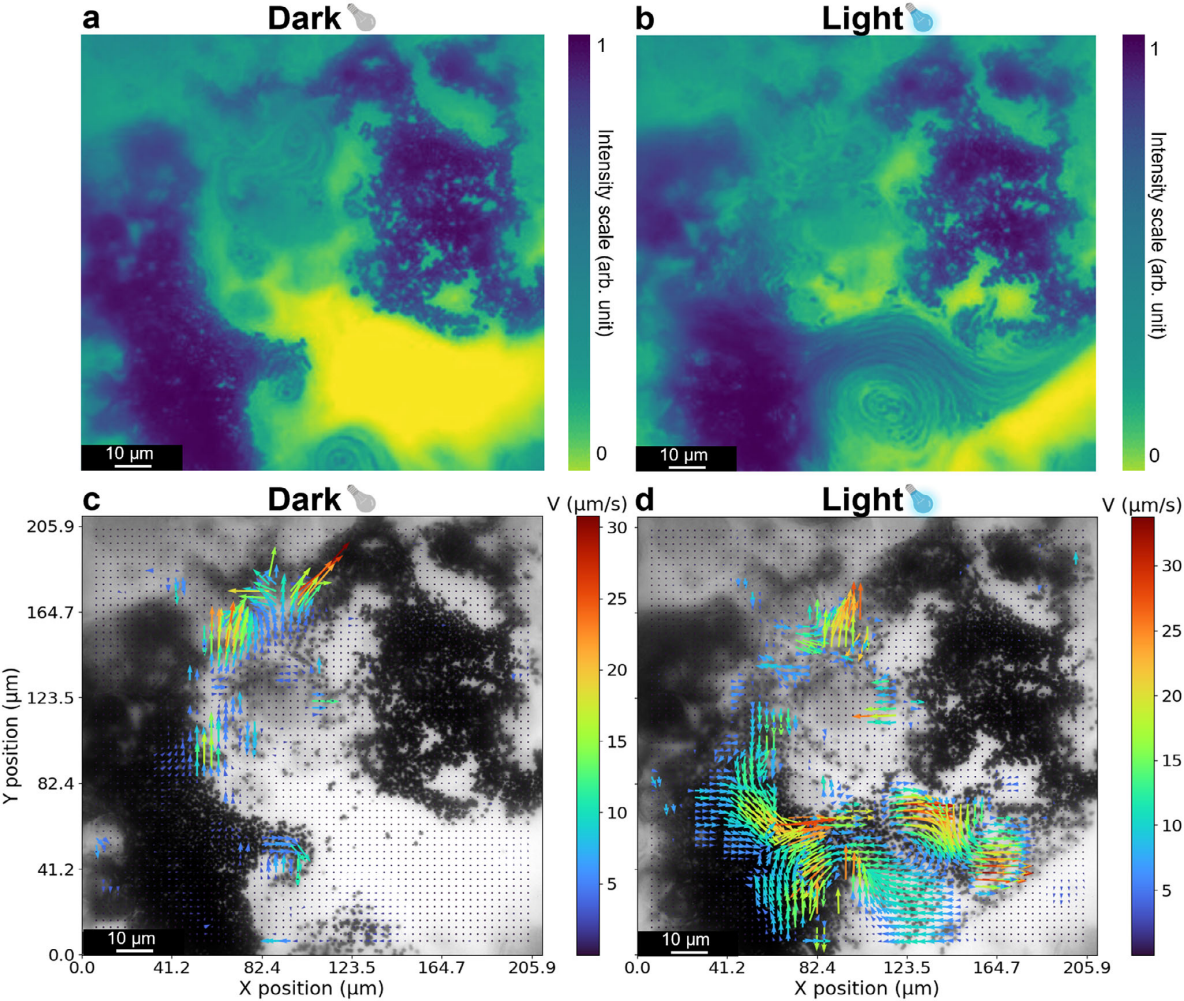
Collective behavior analysis of Cu_2_O micromotors inside a PCLM under dark and light (440 nm) conditions. Density maps (a and b) and PIV analysis (c and d) of Cu_2_O micromotors (0.30 mg mL^−1^ in water). The average pixel intensity was calculated over a 10 s video. PIV analysis indicates the flow field distribution in dark condition (c) and immediately after illumination (d).

Complementary to the single‐particle tracking, we also evaluated the collective behavior of micromotors inside PCLMs. In highly loaded PCLMs, concentrated micromotor suspensions tend to agglomerate and settle near the bottom (Figure S6, Supporting Information), where they generate localized regions of activity together with the intrinsic PCLM flows. Density map analysis (Video S6, Supporting Information; **Figure** **4**a) reveals that, at high loading, Cu_2_O micromotors often form agglomerates that move collectively within localized vortical patterns. Upon illumination (Figure 4b), electrostatic repulsion between the micromotors, due to chemical gradient generation, disrupts these aggregates, leading to a reorganization of the bulk flow into new vortex patterns. PIV analysis (Figure 4c,d) further shows that this light‐induced activity modifies the overall flow field, generating more dynamic circulation and enhancing mass transfer across the entire system.

To further explore the tunability of the PCLM system, we evaluated the collective behavior of BiVO_4_ photoactive micromotors (Video S6 and Figure S7, Supporting Information). In the dark, motion was largely governed by PCLM‐induced flows. Under illumination, stronger repulsive interactions between micromotors disrupted the vortical patterns, producing characteristic radially‐expanded trajectories along the interface. Corresponding PIV data reveals a redistribution of the flow field, with velocities decreasing but spanning a larger fraction of the marble. On the other hand, to compare active and passive transport, we introduced passive PS tracers alongside BiVO_4_ micromotors (Video S7, Supporting Information). While the micromotors exhibit light‐responsive motion distinct from the background flow, passive tracers merely follow the streamlines, confirming that photocatalytic activity provides an additional contribution to micromotor motion.

Building on the analysis of micromotor motion and collective behaviors, we next examined how environmental parameters such as pH and ionic strength affect Cu_2_O micromotors in PCLMs. Video S8 (Supporting Information) compiles observations under acidic and alkaline conditions, both in the dark and under illumination. Under acidic (HCl) or alkaline (NaOH) conditions at low concentrations (1 mm), micromotor motion on glass slides was rapidly quenched. In PCLMs, acidic media caused strong immobilization of micromotors on the silica shell, whereas alkaline media quenched motion on glass but did not induce fixation within the marble. The influence of ionic strength was examined using KCl (0.001–1 m), revealing complete quenching of motion in both glass slides and PCLMs under dark and light conditions. These behaviors can be explained by considering the ζ‐potentials of Cu_2_O micromotors and fumed hydrophobic silica, which are both negative at neutral pH. In acidic media, protonation reduces the negative surface charge of Cu_2_O, promoting electrostatic attraction to the partially immersed silica shell. At high ionic strength, electrostatic screening suppresses phoretic propulsion, leading to the observed quenching.

### 2.4 Photocatalytic Performance of Micromotors in PCLMs

**Figure 5:**
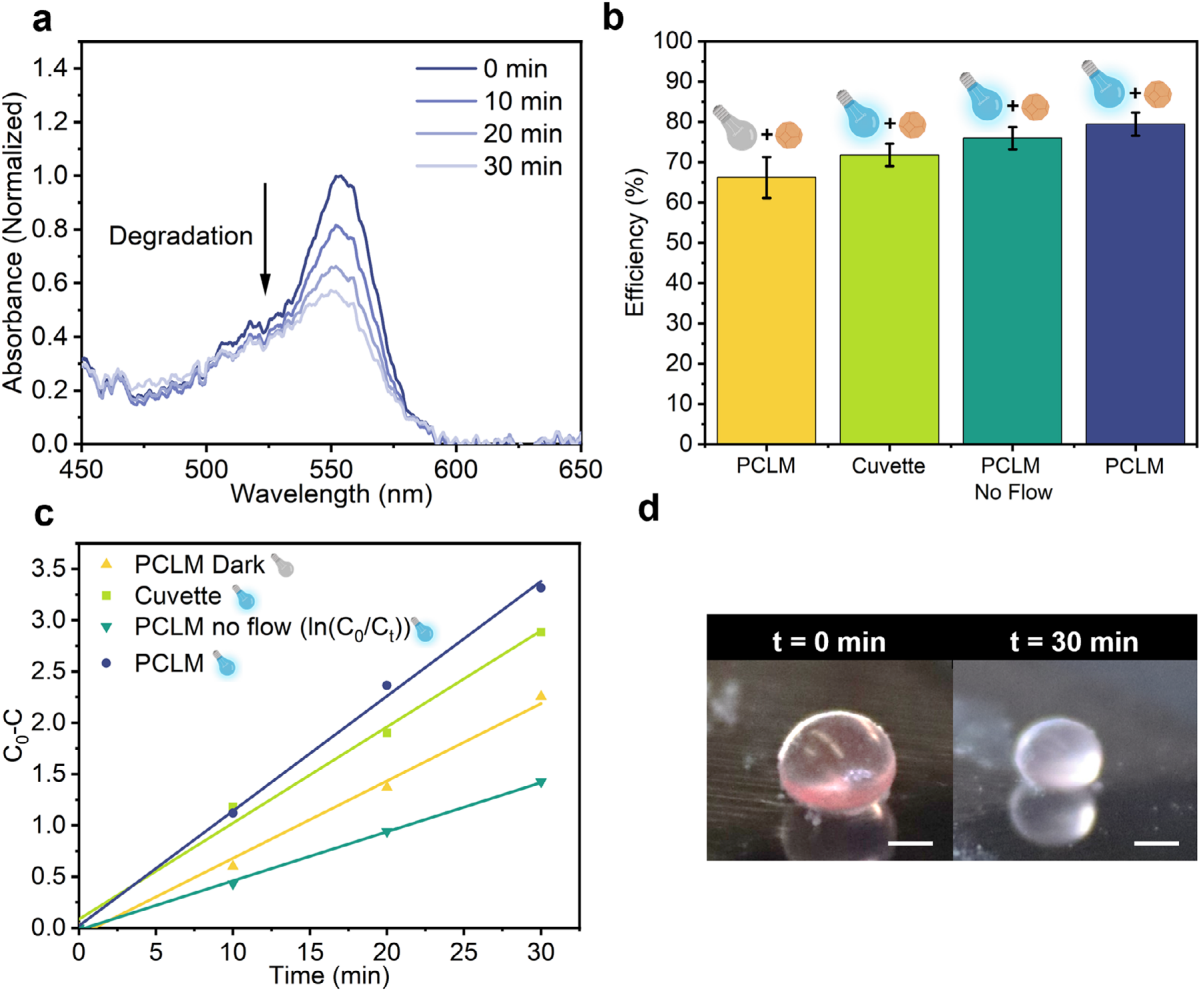
Photocatalytic degradation of RhB by Cu_2_O micromotors inside PCLMs. a) UV–vis absorbance spectra for a PCLM containing Cu_2_O micromotors (0.3 mg mL^−1^), H_2_O_2_ (1%), and RhB (5 ppm) under visible light irradiation (Spectra are normalized to the maximum absorbance at time = 0 min). b) Photocatalytic performance of Cu_2_O micromotors under four different conditions: quartz cuvette (light), PCLM (dark and light), and PCLM in a closed chamber (no flow). Error bars represent standard deviation of the mean, (*n* = 3). c) Reaction kinetics for the same four cases. The “PCLM no flow” condition is plotted as **l**n(C_0_/C_t_) for a better fitting (pseudo‐first reaction order). Reaction rates, R^2^ values, and fitting parameters are provided in Table S2 (Supporting Information). d) Photographs showing the color change of the PCLM before and after 30 min of the photocatalytic reaction. Scale bar: 1 mm.

To determine whether the enhanced micromotor motion in PCLMs leads to improved photocatalytic activity, we tested the degradation of Rhodamine B (RhB) under visible light. For these experiments, PCLMs were loaded with Cu_2_O micromotors, H_2_O_2_ as a chemical fuel, and RhB solution. Reactions were performed in open PCLMs to preserve evaporation‐induced flows, which, as previously demonstrated, promote micromotor mobility and mixing. **Figure** **5**a shows a representative degradation experiment in a PCLM containing Cu_2_O micromotors. In this configuration, the measured absorbance reflects not only photocatalytic activity but also solvent evaporation, which concentrates the dye over time. To disentangle these effects, a series of control experiments were performed (Figure S8a, Supporting Information). The largest apparent concentration increase was observed for PCLMs containing RhB and Cu_2_O without H_2_O_2_, i.e., in the absence of any catalytic reaction (Figure S8b, Supporting Information). The percentage variation from this control was used to correct the photocatalytic efficiencies of all PCLM degradation experiments.

For comparison, the photocatalytic performance of the micromotors was also evaluated in a quartz cuvette under identical concentrations (Figure S8c, Supporting Information). After 30 min, the degradation efficiency of the micromotors in PCLMs reached 79.4%, significantly outperforming an equivalent system in a quartz cuvette, which reached 71.8% under identical conditions (Figure 5b). Notably, Cu_2_O micromotors + H_2_O_2_ in the dark still degraded RhB degradation inside PCLMs (66.2% efficiency) via Fenton‐like reaction, although with lower efficiency than under illumination. Under sealed (closed chamber), no flow conditions, the micromotors inside the PCLM under light irradiation still reached 76.0% efficiency, underscoring the role of flow‐enhanced micromotor mobility in boosting mass transport.

Kinetic analyses for each condition are presented in Figure 5c, with rate constants, reaction orders, and fitting parameters summarized in Table S2 (Supporting Information). The calibration curve used to determine initial RhB concentrations is shown in Figure S8d (Supporting Information). Overall, controlling flow within PCLMs emerges as a promising strategy to further accelerate reaction rates while enabling real‐time, visual monitoring of catalytic progress (Figure 5d).

This work advances the design of low‐volume catalytic systems by integrating light‐powered micromotors into flow‐active, self‐contained microreactors. LMs combine spatial confinement with internal convection, enabling enhanced mass transfer while minimizing reagent use and waste. The gas‐permeable shell supports controlled reagent exchange, positioning PCLMs as promising platforms for studying active matter and MNMs in flow‐active media, confined catalysis, and microscale chemical processing.

From a practical perspective, the scalability and application potential of this approach warrant consideration. Key challenges include preserving LM stability under variable environmental conditions, ensuring long‐term durability of materials, and adapting the platform for continuous‐flow operation. Potential solutions involve employing more robust hydrophobic coatings, developing modular fabrication methods for PCLMs, and integrating them into microreactor arrays. These advances could enable confined photocatalytic systems for micromixer reactors, gas sensing, pollutant remediation, and reaction engineering within lab‐in‐a‐droplet platforms.

## 3 Conclusion

We introduce a soft confinement strategy for enhancing micromotor propulsion and catalytic performance by integrating light‐driven micromotors within LMs. By tuning the shell coverage to obtain PCLMs, robust evaporation‐induced flows arise, increasing the average micromotor velocity by nearly threefold compared to stagnant conditions, primarily due to sustained convection. Under illumination, photocatalytic self‐propulsion contributes an additional velocity component and induces dispersive behavior. This dual driving mechanism promotes directed particle distribution along the liquid–air interface, accelerates mass transport, and enhances photocatalytic degradation efficiency. These findings establish LMs as deformable, flow‐active microreactors for confined photocatalysis and soft active matter studies. In addition to propulsion enhancement, PCLMs facilitated micromotor transport along a gas‐permeable, topographically complex interface while performing photocatalytic reactions. More broadly, this approach opens new directions for exploring collective micromotor behavior, confinement‐guided transport, and biologically inspired dynamics in programmable active systems.

## 4 Experimental Section

### Materials

Copper (II) sulfate pentahydrate 98 + % (Sigma–Aldrich), D‐(+)‐Glucose (Glentham), Sodium hydroxide pellets (Panreac), Hydrophobic Fumed Silica TD‐15 (Guangzhou CE Chemicals Co., Ltd), Polybead polystyrene microspheres aqueous suspension (2.8% w/v, 1.5 µm diameter) (Polysciences), 3‐aminopropyltriethoxylane (APTES) (Sigma–Aldrich), fluorescein isothiocyanate (FITC) (Sigma–Aldrich), Rhodamine B (Serviquimia), H_2_O_2_ 30% (v/v) (Sigma–Aldrich), Absolute ethanol (Sigma–Aldrich), Toluene (Sigma–Aldrich), Methanol (Chemlab), and Mili Q water.

### Synthesis of Cu_2_O Micromotors

Cu_2_O micromotors were prepared via reduction in alkaline media.^[^ ^53^ ^]^ 1.3983 g CuSO_4_·5H_2_O, 360 mL water, and 0.5044 g D‐(+)‐glucose were added to a 500 mL round bottom flask with a magnetic bar stirrer. The mixture was heated to 75 °C while stirring at 700 rpm. 1.25 m NaOH was added dropwise until reaching pH 11.5. Upon NaOH addition, the solution transitioned into a suspension, indicating particle formation. After reaching a constant pH, the suspension was stirred one more hour at room temperature to grow the particles. The suspension was vacuum filtered, washed 3 times with 20 mL deionized water and one time with absolute ethanol. Particles were dried overnight in the oven at 40 °C.

### Liquid Marble Formation and Loading

Hydrophobic fumed silica powder was added to a petri dish until a powder bed was formed. Then, a 5 µL (for motion characterization) or 10 µL (for photocatalytic degradation) droplet of the colloidal suspension (PS tracers or Cu_2_O motors) was added to the silica bed and rolled until its surface was completely coated. A quick rolling process was made to obtain PCLMs, and an extensive rolling provided CCLMs. For further analysis the marbles were cleaned gently by blowing air with a Pasteur pipette and transferred with a micro‐spatula.

### Fumed Silica Fluorescent Labeling

Fluorescent labeling was performed in two steps. First, 2.0 g Hydrophobic Fumed Silica TD‐15 particles and 0.002 mmol 3‐aminopropyltriethoxylane (APTES) were added in to a 40 mL toluene. This mixture was heated to 60 °C, under N_2_ atmosphere and stirred for 2 h. Functionalized particles were centrifuged, washed three times with 20 mL toluene and two times with 10 mL ethanol. The solid was dried in oven at 40 °C overnight. Second, 0.5 g of functionalized fumed silica particles (produced in step 1), and 0.005 g fluorescein isothiocyanate (FITC) were added in 50 mL absolute ethanol in a glass beaker and stirred overnight at room temperature. Finally, fluorescently labeled fumed silica particles were collected by centrifugation and washed three times with 10 mL ethanol and one time with 10 mL methanol and dried under vacuum.

### Characterization of LMs

The surface morphology of LMs was examined by an environmental emission scanning electron microscope with a focused Ga ion beam (ESEM‐FIB, Scios 2 by FEI Company). Hydrophobic fumed silica particles were also characterized by transmission electron microscope (TEM) JEOL 1011 of 100 Kv and tungsten filament. The powder XRD data were obtained using a Bruker AXS D8‐Discover diffractometer (40 kV and 40 mA). Particle size and Zeta potential were determined by Dynamic Light Scattering (DLS) on a Malvern ZEN3600 NanoZS apparatus equipped with a 633 nm HeNe laser. Thermal imaging was conducted using an M11 HIKMICRO thermal camera.

### Motion Characterization

Flow characterization was performed by tracking 20 PS tracers (used as passive tracers) within the LMs. A 5 µL droplet of the PS tracers suspension was deposited onto a bed of hydrophobic fumed silica and rolled to form PCLMs and CCLMs. These LMs were then transferred to a glass slide and imaged using a THUNDER Imager Modular DMi8 inverted microscope (Leica) the focal plane for all the videos is indicated in Figure S9 (Supporting Information). Videos were processed using Leica Application Suite X (LAS X) software and tracked with Image J tracking script to estimate flow velocities. For intensity mapping, a PCLM loaded with a suspension of PS tracers (1.5 µm, 0.28% w/v) was transferred to the inverse microscope and recorded. From the recorded videos, 10 s segments (150 frames each) were extracted. These stacks were analyzed using z‐projection by sum slices. The cumulative pixel intensity of these segments was computed and visualized using the viridis lookup table from ImageJ. The PIV analysis of recorded videos was performed using a custom Python code based on the OpenPIV library. Consecutive frames within one s were extracted and processed with an interrogation window size of 64 × 64 pixels (width × height), 16 × 16 pixels overlap (horizontal × vertical), and a frame rate of 15.27 fps. The resulting data was then processed with a Python script to adjust arrow dimensions and visualize color‐coded velocities.

Individual particle tracking, intensity mapping, and PIV for Cu_2_O were analyzed with a similar method. For each condition, 20 individual micromotors were tracked. Experiments were performed both on glass slides and within 5 µL PCLMs, under 440 nm light irradiation and in the dark. For glass slide experiments, a 5 µL droplet of a 0.03 mg mL^−1^ aqueous Cu_2_O suspension was deposited directly onto the slide and imaged under inverted microscopy, with light irradiation from below. For marble experiments, a 5 µL droplet of the same Cu_2_O suspension was rolled on a fumed silica bed to form a LM, which was then transferred to a glass slide. All marbles were recorded within the first 10 minutes after the rolling process. A 0.3 mg mL^−1^ suspension of Cu_2_O was also loaded in PCLMs for intensity mapping and PIV.

Ionic strength experiments were carried out by suspending 0.03 mg mL^−1^ Cu_2_O micromotors in KCl 1, 0.1, and 0.01 m to further analyze on a glass slide and PCLM. For pH experiments, the same concentration of Cu_2_O micromotors was prepared in HCl 1, 10, and 100 mm for acidic conditions and NaOH 1, 10, and 100 mm for alkaline conditions.

### Statistical Analysis

All videos were exported from the microscope software in. avi format for further processing, and UV–vis spectra were baseline‐corrected prior to quantification. MSD and kinetic fittings are reported with their respective fitting errors, with numerical values provided in the Supporting Information. Error bars in all column graphs represent the standard error of the mean (SEM) from *n* = 3 independent experiments. MSD from individual particle tracking was calculated from *n* = 20 particles, pathlength tracking from *n* = 13 particles, and degradation efficiency and evaporation controls from *n* = 3 experiments. Fitting errors and general parameters were computed using Origin software, and efficiency calculations were performed using a standard spreadsheet.

## Conflict of Interest

The authors declare no conflict of interest.

## Supporting information



Supporting Information

Supplemental Video 1

Supplemental Video 2

Supplemental Video 3

Supplemental Video 4

Supplemental Video 5

Supplemental Video 6

Supplemental Video 7

Supplemental Video 8

## Data Availability

The data that support the findings of this study are available in the supplementary material of this article.
