# Supplementary material for: Flow‐Active Liquid Marbles as Microreactors for Photocatalytic Micromotors
Source: Small. 2025 Sep 12;21(43):e05439. doi: 10.1002/smll.202505439 (PMC12571213; doi:10.1002/smll.202505439)
Supplement: Supplementary file 1 — Supporting Information [file SMLL-21-e05439-s004.docx]

Supporting Information

Flow-Active Liquid Marbles as Microreactors for Photocatalytic Micromotors

Anthony Jesús Martínez, Majid Basharat, Shuqin Chen, Samuel Sánchez, Katherine Villa*

A. J. Martínez, M. Basharat, K. Villa

Institute of Chemical Research of Catalonia (ICIQ-CERCA), The Barcelona Institute of Science and Technology (BIST), Av. Països Catalans, 16, Tarragona E-43007, Spain.

E-mail: kvilla@iciq.es

A. J. Martínez

Departament de Química Física i Inorgànica, Universitat Rovira i Virgili, Marcel.lí Domingo 1, 43007, Tarragona, Spain.

S. Chen, S. Sánchez.
Institute for Bioengineering of Catalonia (IBEC), The Barcelona Institute for Science and Technology (BIST), Baldiri i Reixac 10-12, Barcelona 08028, Spain.

S. Sánchez.
Catalan Institute for Research and Advanced Studies (ICREA), Psg. Lluis Companys 23, 08010, Barcelona, Spain.

**Liquid marble confinement and structural characterization**

Transmission electron microscopy (TEM) revealed that the silica particles possessed irregular morphologies and a broad size distribution from 5 to 30 nm (Figure S1). Powder X-ray diffraction (XRD) confirmed their amorphous nature, in agreement with known patterns for fumed silica (Figure S2). Dynamic light scattering (DLS) measurements of the silica particles showed a zeta potential of –18.3 mV, reflecting a negative surface charge.


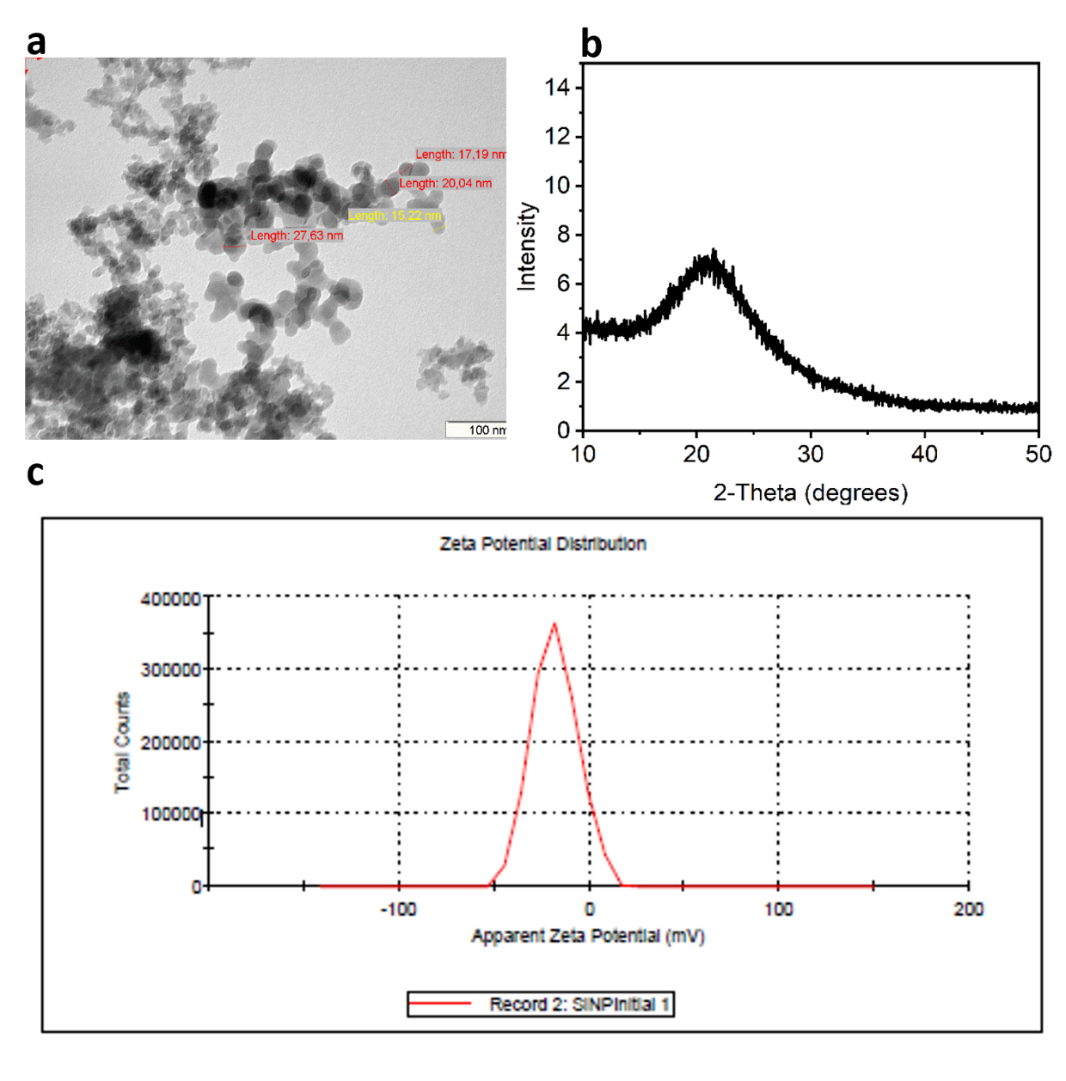


**Figure S1.** Hydrophobic fumed silica characterization. (**a**) TEM image of hydrophobic fumed silica T15 used for coating LMs, revealing aggregates of irregularly shaped nanoparticles with individual lengths ranging from 15 to 28 nm. (**b**) X-ray diffraction pattern of hydrophobic fumed silica T15. (**c**) Zeta potential distribution plot for hydrophobic fumed silica. Peak maximum in -18.3 mV.


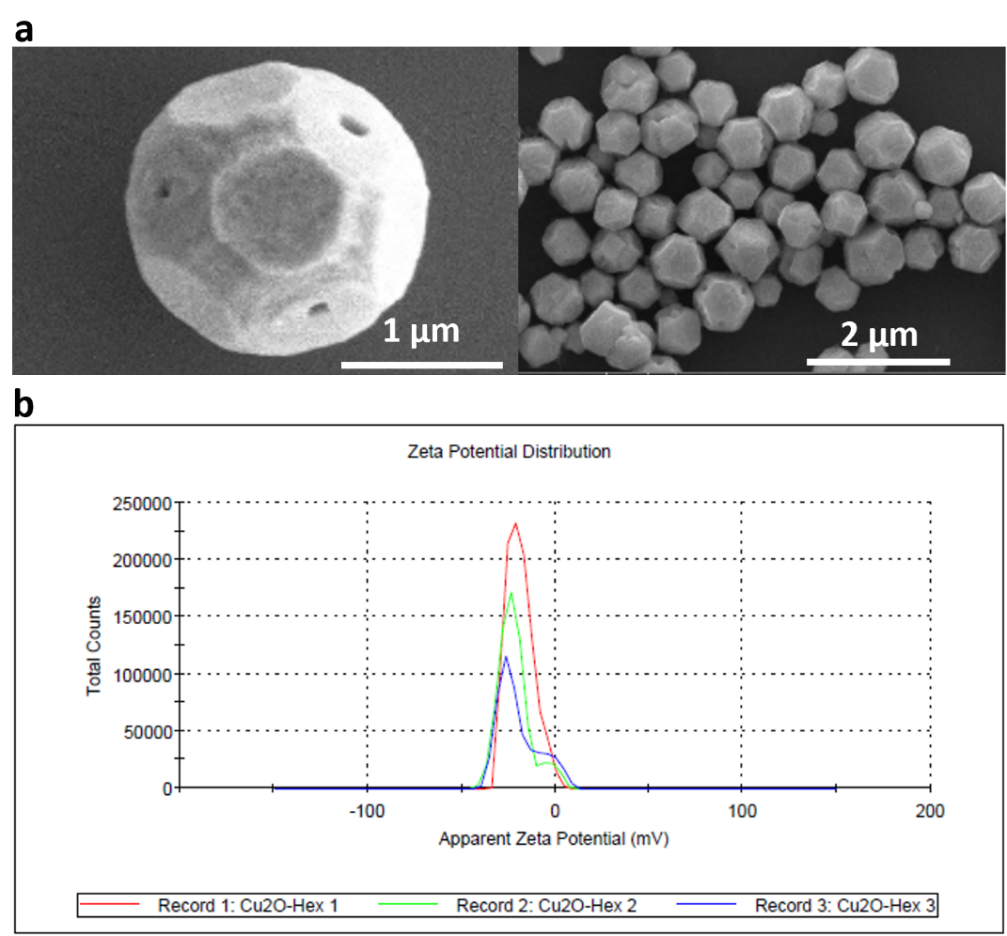


**Figure S2.** Cu_2_O micromotors characterization. (**a**) Field Emission Scanning Electron Microscope (FESEM) images of Cu_2_O micromotor. Average particle size 1.6 µm. (**b**) Zeta potential distribution plot for Cu_2_O micromotors. Peak maximum at -18.7 mV.


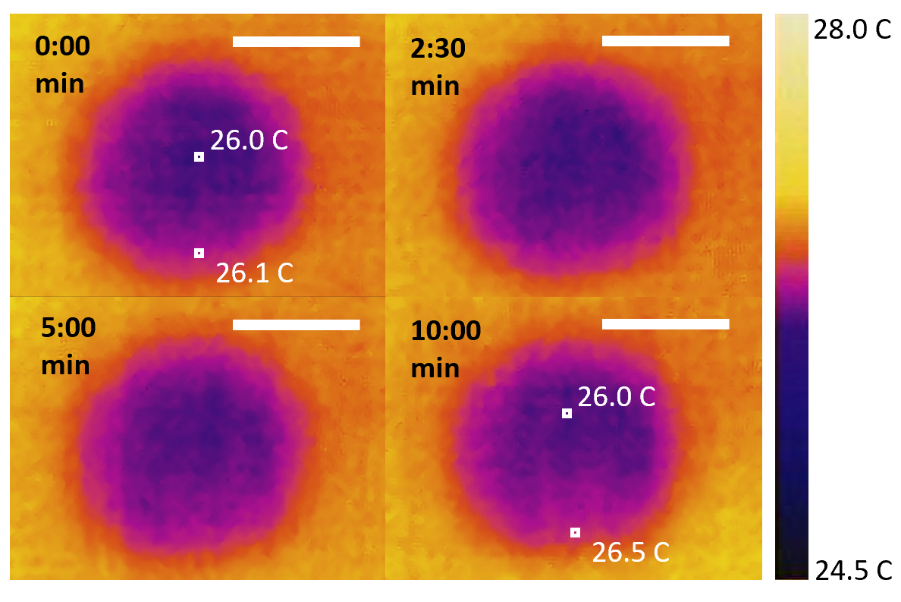


**Figure S3.** Top view thermographic images of a 10 µL PCLM under ambient conditions over a 10 min period. Scale bar: 1.0 mm.


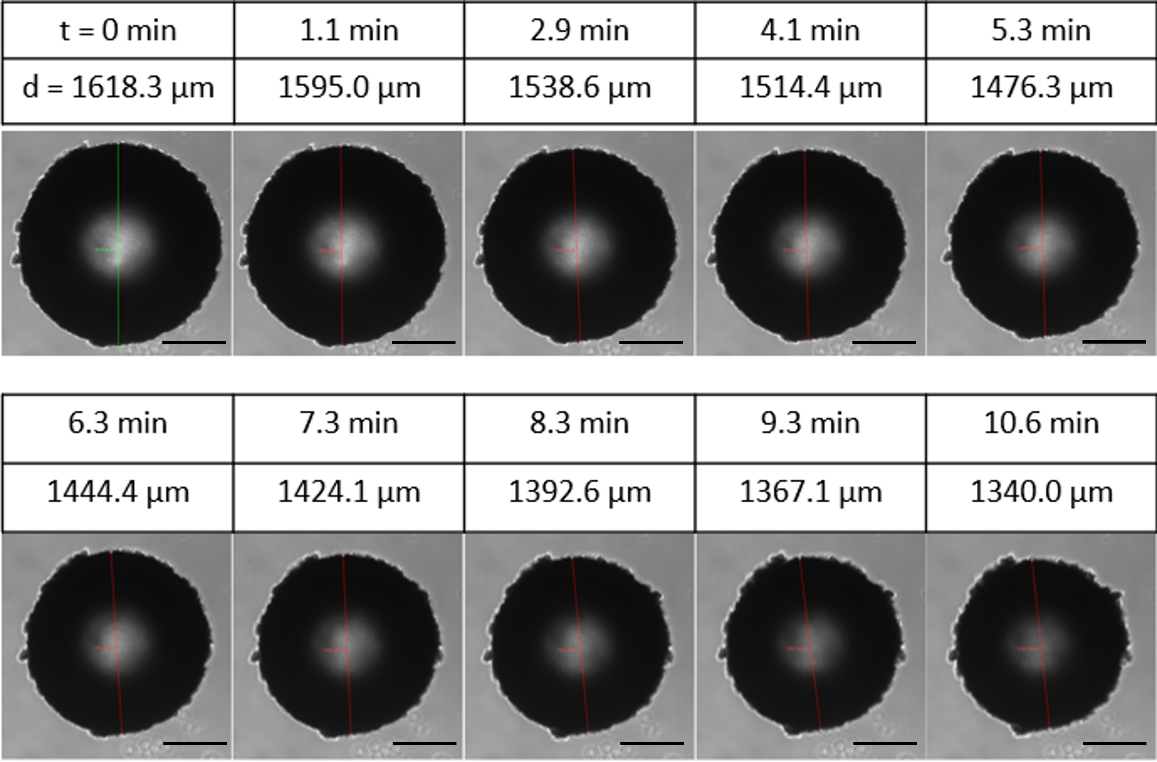


**Figure S4.** Time-lapse optical images showing the progressive diameter reduction of a 5 µL partially covered liquid marble (PCLM) over 10.6 minutes under ambient conditions. The measured diameters at each time point reflect evaporation-driven shrinkage, which was used to estimate the characteristic length (L) for flow regime analysis. Scale bar: 0.5 mm.

**Evaporation-induced microflows in LMs**

**Table S1.** Parameters from MSD quadratic fitting for individual tracking of polystyrene tracer particles (n=20).

| Fitting equation | MSD = 4D*t + v^2*t^2 | |
| --- | --- | --- |
|  | **Value** | **Error of the mean** |
| D | 3.6545 | 0.6407 |
| V | 15.0908 | 0.9430 |
| V^2^ | 227.7311 | 28.2178 |
| Square-R | 0.9994 |  |


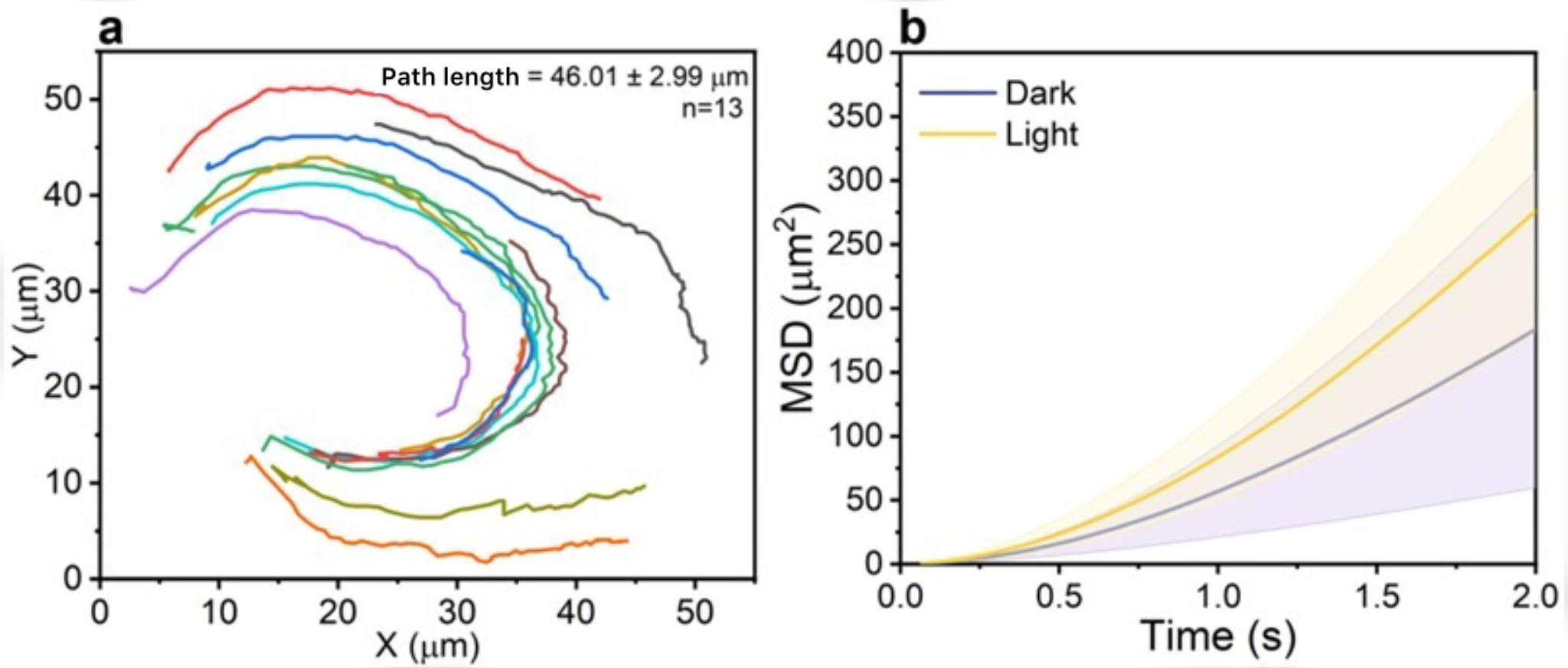
**Micromotor motion dynamics within LMs**

**Figure S5.** Single-particle tracking over a 10 s video in a selected area, where *n*=13 particles display overlapping trajectories**.** (**a**) Trajectory plot with the corresponding average path length. (**b**) MSD) under dark and light conditions.


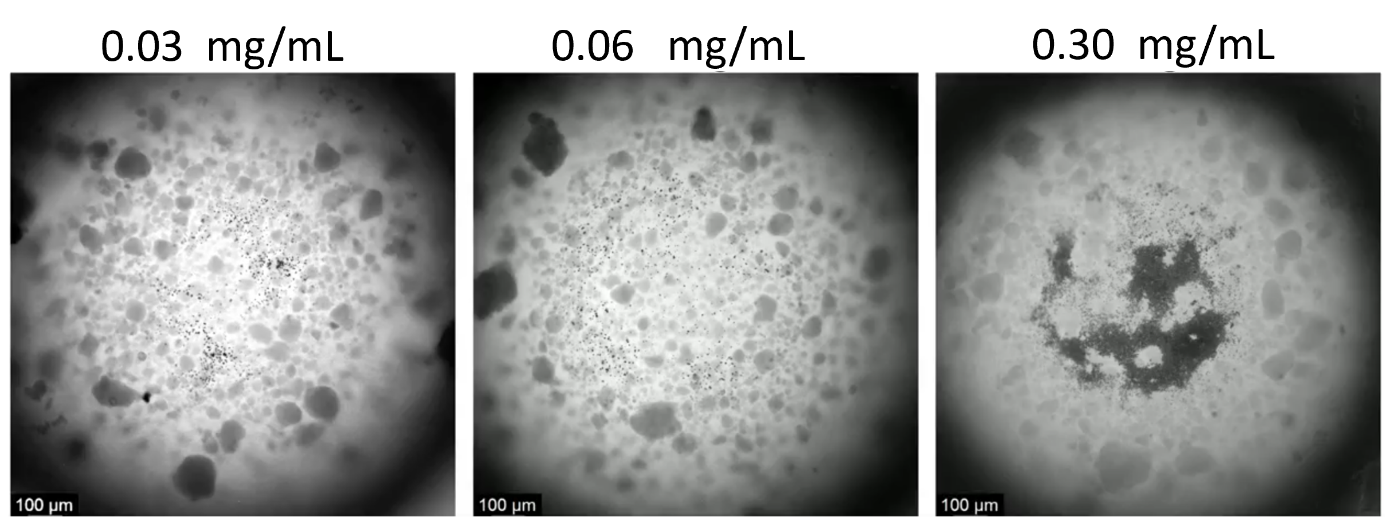


**Figure S6.** PCLMs loaded with different concentrations of Cu_2_O micromotors. 0.03 and 0.06 mg/mL images show the micromotors as little black dots dispersed in the visualized area while 0.3 mg/mL image shows that the micromotors are agglomerated at the bottom interface (black agglomerates in the center).


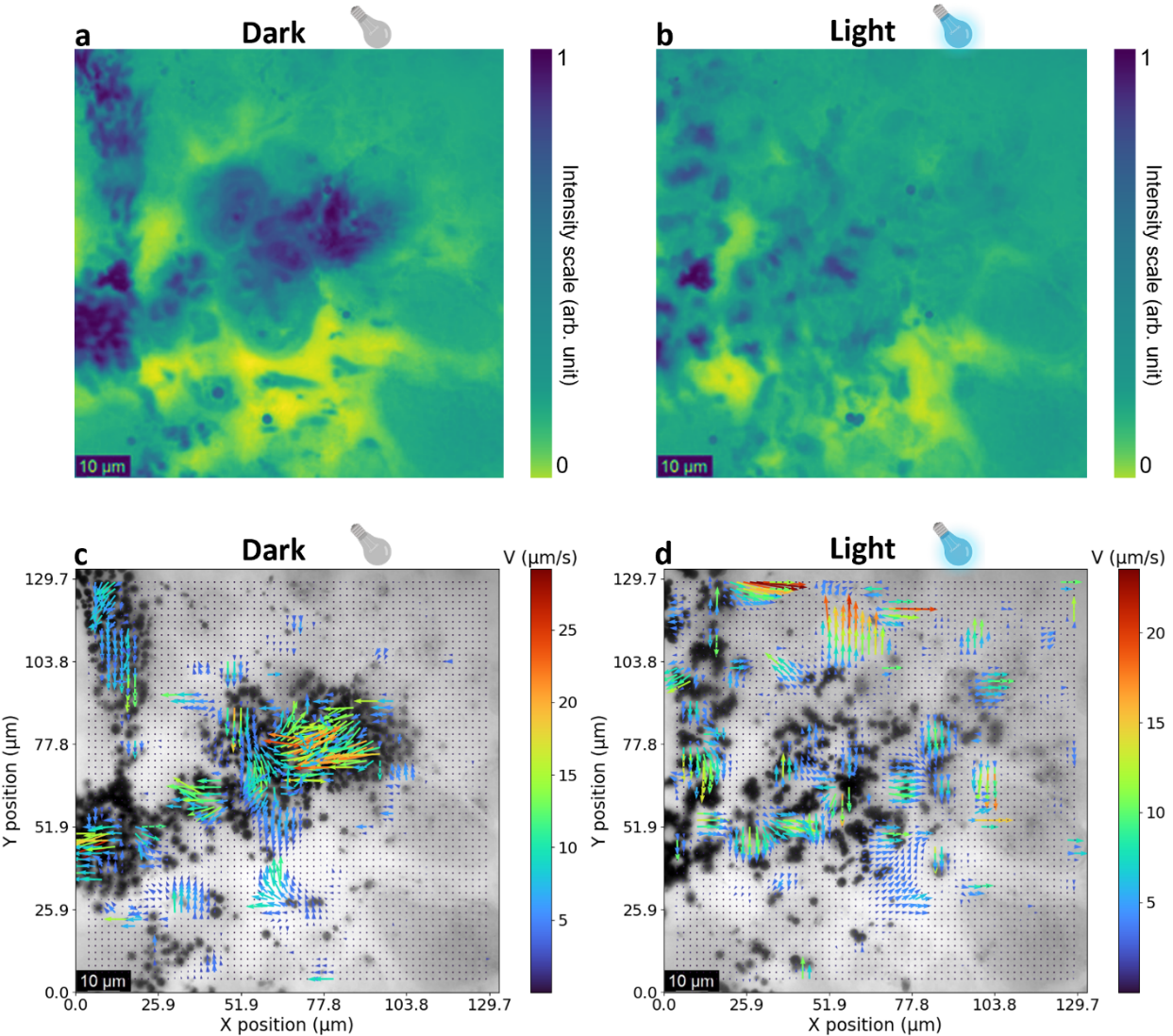


**Figure S7.** Collective behavior of BiVO_4_ micromotors inside a PCLM under 440 nm illumination and dark conditions. (**a, b**) Intensity maps obtained by averaging pixel intensity over a 10 s video. (**c, d**) particle image velocimetry (PIV) analysis showing the flow field distribution in dark condition (c) and immediately after illumination (d). All experiments were performed with BiVO_4_, at a 0.30 mg/mL concentration, in the presence of 1% H_2_O_2_ as chemical fuel.

**Photocatalytic performance of micromotors in PCLMs**


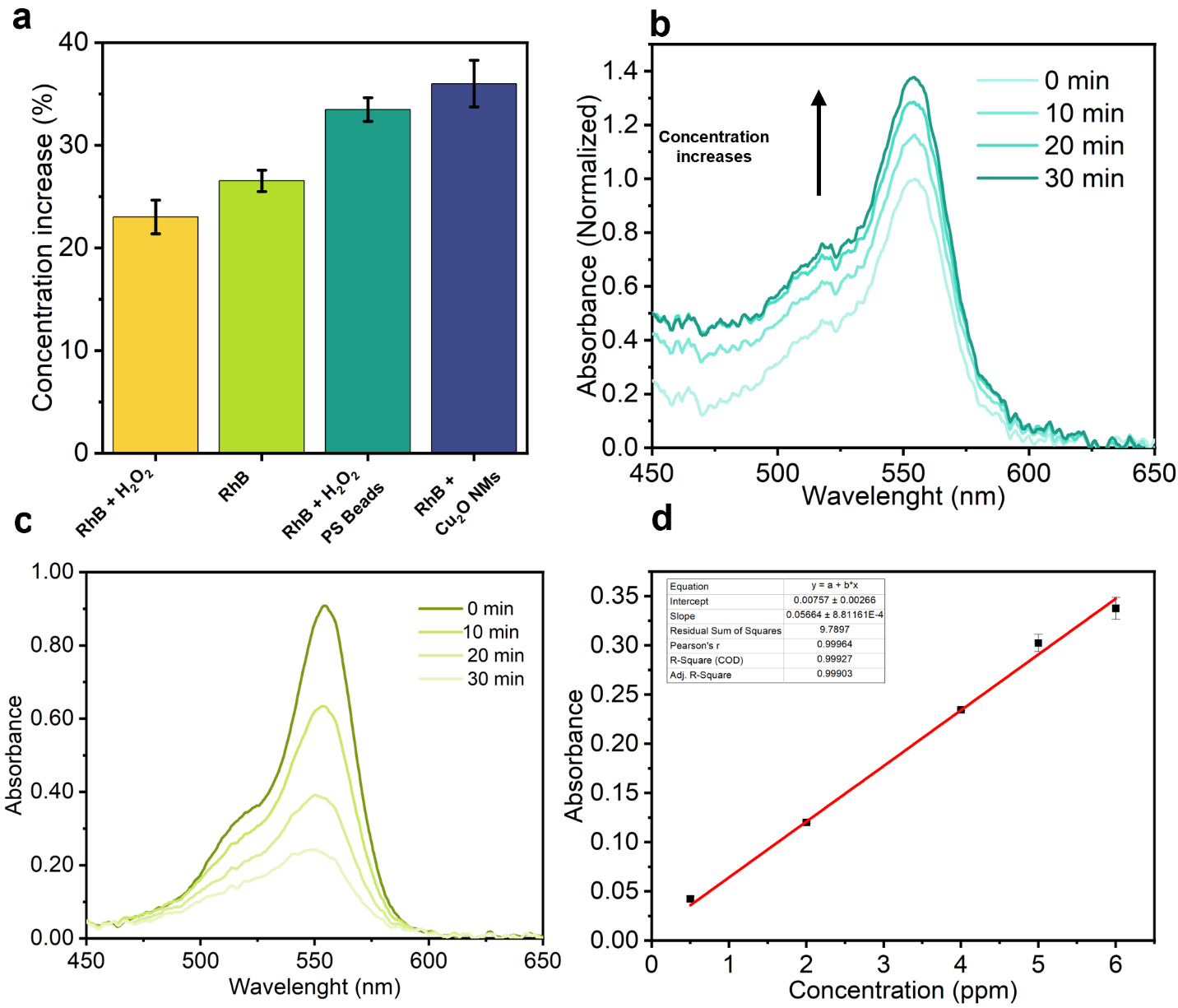


**Figure S8.** Supporting data of the spectroscopic analysis of controls, including Rhodamine B, H_2_O_2_ and Cu_2_O micromotors system within PCLMs. **a.** Evaporation control experiments showing concentration increase over time as a function of PCLM composition. **b.** UV-vis absorbance spectra of the control of Rhodamine B + Cu_2_O micromotors measured to estimate the effect of PCLM evaporation on concentration increase (spectra normalized to absorbance at t = 0 min to 1.0 a.u.). **c.** UV–vis absorbance spectra recorded during the photocatalytic degradation of Rhodamine B (5 ppm) in a conventional quartz cuvette containing Cu_2_O micromotors (0.30 mg/mL) and H₂O₂ (1%) under visible light irradiation. **d.** Calibration curve and fitting parameters for PCLMs loaded with Rhodamine B at time = 0 min.

**Table S2**. Fitting parameters for the reaction kinetics of Rhodamine B (5 ppm) degradation in PCLMs containing Cu_2_O micromotors (0.30 mg/mL) and 1% H_2_O_2_.

| **Case** | **Cuvette** | **PCLM** | **PCLM Dark** | **PCLM No Flow** |
| --- | --- | --- | --- | --- |
| Fitted plot | C_0_-C vs Time | | | Ln(C_0_/C) vs Time |
| Reaction order | Zeroth Order | | | Pseudo First Order |
| Slope (k) | 0.09373 ± 0.00598 | 0.11193 ± 0.00398 | 0.07533 ± 0.0045 | 0.04786 ± 0.00113 |
| Pearson's R | 0.99596 | 0.99874 | 0.99645 | 0.99944 |
| R-Square | 0.99193 | 0.99747 | 0.99291 | 0.99889 |

***Note:*** *All the videos were taken in the bottom interface (focal plane* ***c*** *in* ***Figure S9****) with an inverted microscope.*


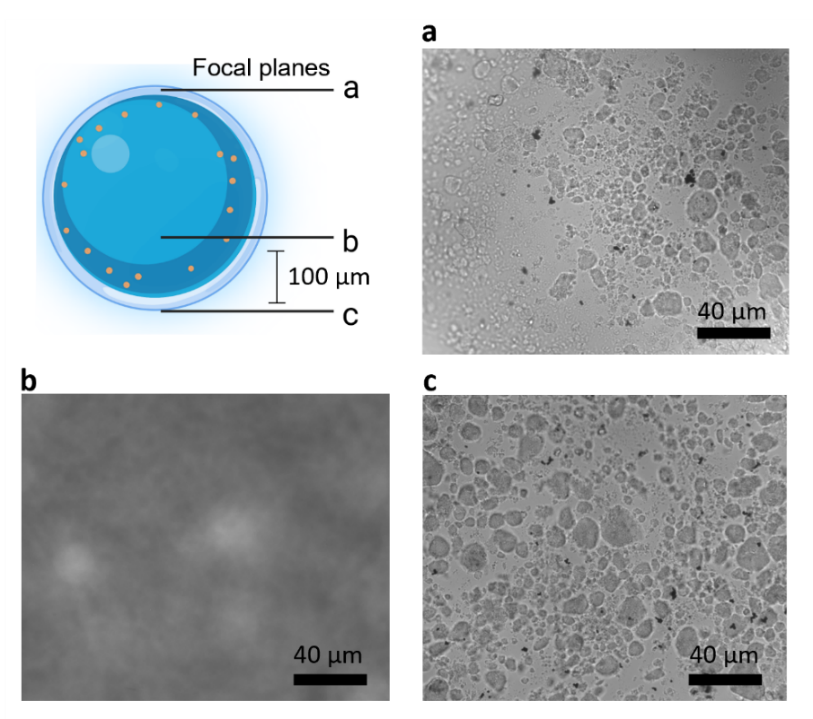


**Figure S9**. Focal planes and motors distribution in PCLMs. (**a**) Top interface, (**b**) 100 µm from bottom interface and (**c**) bottom interface.

**Supplementary videos**

**Video S1.** Comparison of a completely covered liquid marble (CCLM) and a partially covered liquid marble (PCLM), each loaded with 1.5 µm polystyrene beads (0.028 w/v %). Recordings were made at the bottom interface. Particle motion occurs only in PCLMs.

**Video S2.** PCLM loaded with 1.5 µm polystyrene beads (0.028 w/v %). In an open environment, particles move due to evaporation-induced flows. In a closed chamber, motion ceases due to suppression of evaporation.

**Video S3.** 3D Z-stack reconstruction of a PCLM containing 1.5 µm fluorescent beads (0.3 mg/mL). The bottom section (Z = 0–160 µm) shows particles distributed uniformly along the interface but absent in the PCLM interior.

**Video S4**. Typical behavior of Cu_2_O micromotors on a glass slide under 440 nm light irradiation.

**Video S5**. PCLM containing 0.03 mg/mL Cu_2_O micromotors under 440 nm light irradiation. Motion is recorded at the bottom focal plane

**Video S6**. PCLMs containing either Cu_2_O micromotors (0.3 mg/mL) and BiVO_4_ micromotors (0.3 mg/mL). Light on/off cycles reveal collective behaviors arising from the interplay of photoactivity and flow-induced transport

**Video S7**. PCLM containing a mixture of polystyrene beads (0.28 mg/mL) and BiVO_4_ micromotors (0.30 mg/mL) in water. Light on/off cycles show that BiVO_4_ micromotors are activated by light, while polystyrene beads remain passive.

**Video S8**. Effect of pH on Cu_2_O micromotor activity. Suspensions of micromotors (0.03 mg/mL) in 1 mM HCl or 1 mM NaOH were tested inside PCLMs and on glass slides. HCl promotes micromotor adhesion to both substrates, whereas NaOH quenches motion on glass slides but not inside PCLMs. *Note: Since a manual shutter was used to open the light beam, some shaking is observed before illumination begins.*
